# Study on the ultrasonic myocardial mechanics of left atrioventricular coupling in IBSH patients

**DOI:** 10.3389/fcvm.2025.1646874

**Published:** 2026-02-11

**Authors:** Huimin Jia, Ting Ma, XiaoMei Hu, Jing Nan, Shi Qi, Yuming Mu, Lina Guan

**Affiliations:** 1 Department of Echocardiography, First Affiliated Hospital of Xinjiang Medical University, Urumqi, China; 2 Key Laboratory of Ultrasound Medicine of Xinjiang, Urumqi, China; 3 State Key Laboratory of Pathogenesis, Prevention and Treatment of High Incidence Diseases in Central Asia, First Affiliated Hospital of Xinjiang Medical University, Urumqi, China

**Keywords:** hypertension, IBSH, left atrial strain, left atrioventricular coupling, left ventricular strain

## Abstract

**Objective:**

This study aimed to evaluate the myocardial mechanical properties of the left atrium, left ventricle, and left atrioventricular coupling using ultrasound in patients with isolated basal septal hypertrophy (IBSH) caused by primary hypertension.

**Methods:**

This retrospective study enrolled 210 participants [140 hypertensive patients subdivided into IBSH and left ventricular hypertrophy (LVH) groups (*n* = 70 each) and 70 controls]. Speckle-tracking echocardiography was used to analyze left atrium (LA)/left ventricle (LV) strain parameters and atrioventricular coupling.

**Results:**

(1) The IBSH group demonstrated higher adherence to antihypertensive medication than the LVH group (*P* < 0.05). (2) Both the IBSH and LVH groups showed reduced global longitudinal strain (GLS) and global circumferential strain (GCS) (more severe in LVH) but increased global radial strain (GRS) (more prominent in IBSH) (*P* < 0.05). The 18-segment LV strain analysis revealed significantly reduced strain in the basal anterior and inferior septum (*P* < 0.05), while compensatory increased strain in all apical segments (*P* < 0.05) in the IBSH group. (3) Left atrioventricular coupling index (LACI) increased in both groups (*P* < 0.01), while left atrial reservoir strain (LASR) and left atrial conduit strain (LASCD) decreased (more in LVH), yet left atrial contraction strain (LASCT) markedly rose in IBSH (*P* < 0.01). (4) Correlation analysis in the IBSH group showed the following results: Left ventricular mass index (LVMI) negatively correlated with left ventricular ejection fraction (LVEF), GRS, and GCS (*P* < 0.05); LACI positively correlated with left atrial volume index(LAVI) (*r* = 0.73); LASR positively correlated with GLS and E/A but negatively with E/e′ (all *P* < 0.05).

**Conclusion:**

This study elucidates the potential association between regular antihypertensive medication use and cardiac remodeling in patients with IBSH. Through a comprehensive analysis of global and regional left atrioventricular myocardial function, we identified the basal septal segment as the most significantly impaired myocardial region. Furthermore, the study demonstrated the existence of a compensatory redistribution mechanism in myocardial mechanics.

## 1 Background

Previous studies have considered isolated basal septal hypertrophy (IBSH) a degenerative change associated with aging; however, its prevalence in hypertensive patients is as high as 20% (1). Earlier research has shown that in patients with hypertension, increases in wall thickness occur gradually and unevenly. Wall stress is influenced by the local geometry of the LV, with areas having a larger curvature radius exposed to higher wall stress (2, 3). Compared with the free wall, the basal segment has a larger curvature radius (2). As blood pressure increases, wall stress rises disproportionately, resulting in progressive thickening from the apical to the basal segment. According to Laplace's law, an imbalance between increased wall stress and locally generated forces leads to reduced local deformation. Over time, this imbalance may cause localized hypertrophy, which is considered a key mechanism underlying IBSH (4). However, the effects of this LV remodeling on the left atrium (LA) and atrioventricular coupling remain poorly understood (5). Therefore, this study aimed to explore the myocardial mechanical relationships among the LA, LV, and left atrioventricular coupling in the IBSH group.

This study utilizes two-dimensional speckle-tracking imaging (2D-STI) to assess the myocardial function of the left atrium and left ventricle. By identifying and tracking myocardial motion on echocardiographic images, 2D-STI enables quantitative analysis of myocardial strain (6). Parameters including GLS, GRS, global circumferential strain (GCS), and 18-segment left ventricular strain are derived echocardiographically to quantify both regional and overall left ventricular myocardial motion, thereby providing a systematic evaluation of left ventricular myocardial function. In recent years, increasing attention has been directed toward the assessment of left atrial function using the same 2D-STI methodology (7). Accordingly, this study aims to analyze the myocardial mechanical characteristics and intrinsic relationships of the left atrium, left ventricle, and left atrioventricular coupling in patients with IBSH.

## 2 Content and methods

### 2.1 Study subjects

A total of 140 hypertensive patients diagnosed at the First Affiliated Hospital of Xinjiang Medical University between July 2023 and May 2024 were enrolled in the case group. Based on baseline data, patients were classified into the IBSH group (*n* = 70) and the left ventricular hypertrophy (LVH) group (*n* = 70), according to the location of septal hypertrophy. An additional 70 healthy individuals were included as the control group. This study was conducted in strict accordance with the principles of the Declaration of Helsinki and has been reviewed and approved by the Ethics Committee of the First Affiliated Hospital of Xinjiang Medical University. As this is a retrospective study, the requirement for written informed consent was waived.

### 2.2 Inclusion and exclusion criteria

Inclusion criteria: Hypertension was defined as systolic blood pressure (SBP) ≥ 140 mmHg or diastolic blood pressure (DBP) ≥ 90 mmHg, measured over three consecutive days. Patients with a history of hypertension and on antihypertensive medications were included even if their blood pressure was below 140/90 mmHg. During echocardiographic examination, blood pressure was required to be controlled below 140/90 mmHg in both groups.

**Figure 1:**
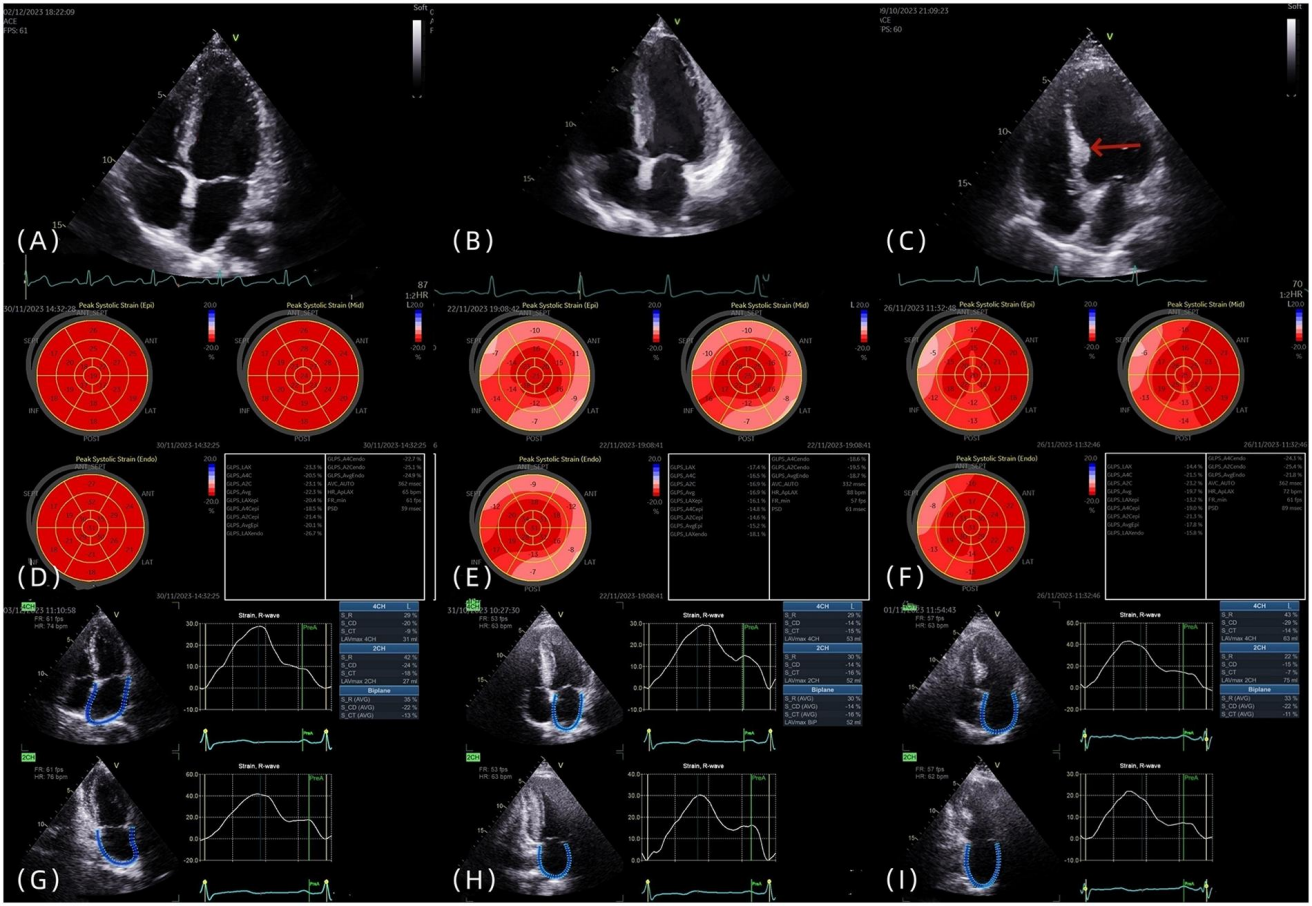
**(A–C)** The four-chamber heart views of the control group, LVH group, and IBSH group, respectively, with the arrow pointing to the posterior septal basal segment of the IBSH group. **(D–F)** The left ventricular strain images of the control group, LVH group, and IBSH group, respectively. **(G–I)** The left atrial strain images of the control group, LVH group, and IBSH group, respectively.

IBSH group: The Framingham inclusion criteria were used as a reference. (1) Localized thickening of the basal septal segment was defined as left ventricular septal basal-segment end-diastolic thickness (LVSBD) ≥ 1.4 cm. (2) The ratio of LVSBD to interventricular septum end-diastolic thickness (IVSD) was required to be ≥1.5. (3) No scarring or segmental wall motion abnormalities were present in the mid-septal segment. (4) Conditions such as aortic stenosis, subaortic membrane, and hypertrophic cardiomyopathy were excluded. All four criteria were required to be met simultaneously (Figures 1A–C).

LVH group: Diagnostic criteria were based on current guidelines (8). Males were required to have left ventricular mass index (LVMI) > 115 g/m² and relative wall thickness (RWT) > 0.42, whereas females were required to have LVMI > 95 g/m² and RWT > 0.42.

Additionally, 70 healthy individuals were enrolled as the control group.

Exclusion criteria: (1) patients with confirmed cardiomyopathy, coronary artery disease, valvular heart disease, or other significant cardiac conditions; (2) those with secondary hypertensive heart disease caused by diabetes, kidney disease, or other systemic conditions; and (3) those with poor echocardiographic image quality.

### 2.3 Clinical data, equipment, and examination methods

#### 2.3.1 Equipment

Two experienced echocardiographers, blinded to patient group allocation, obtained all images using the Vivid E9 ultrasound system (GE, Vingmed Ultrasound, Horten, Norway) with an M5S probe operating at 3.5–5.5 MHz and a frame rate of 50–80 frames per second (fps). Continuous images from five consecutive cardiac cycles were recorded. Offline image analysis was performed using the EchoPAC workstation (GE Medical Systems, version 204.0).

#### 2.3.2 Transthoracic echocardiography

All patients were examined in the left lateral decubitus position following the guidelines of the American Society of Echocardiography (ASE) and the European Association of Cardiovascular Imaging (EACVI) (9).

In the transthoracic left ventricular long-axis view, measurements were taken for the IVSD, LVSBD, and the left ventricular posterior wall end-diastolic thickness (LVPWd). Based on the above measurements, the LVMI and LAVI were subsequently calculated. All parameters were recorded as the average of measurements taken over three consecutive cardiac cycles. From the apical four-chamber view, pulsed-wave Doppler was used to assess mitral inflow velocities, including early diastolic velocity (E), late diastolic velocity (A), and the E/A ratio. Tissue Doppler imaging (TDI) from the apical four-chamber view was used to measure septal annular velocity (e′), and the E/e′ ratio was then calculated.

The following parameters were calculated as described in (10). Left ventricular mass (LVM) was calculated using the corrected formula: LVM = 0.8 × {1.04[(LVDd + LVPWd + LVSd)³ − LVDd³]} + 0.6. LVMI was calculated as LVM ÷ Body Surface Area (BSA). RWT was calculated as (LVPWd + LVSd) / LVDd. LAVI was calculated as LAVmax ÷ BSA.

The biplane Simpson's method was applied in the apical four-chamber and two-chamber views to measure the following parameters: left ventricular end-systolic volume (LVESV), left ventricular end-diastolic volume (LVEDV), left ventricular ejection fraction (LVEF), left atrial maximum volume (LAVmax), left atrial minimum volume (LAVmin), left atrial pre-contraction volume (LAVpreA), left atrial total ejection fraction (LATEF), and left atrioventricular coupling index (LACI). LACI is defined as the ratio of left atrial end-diastolic volume to left ventricular end-diastolic volume.

The following parameters were calculated using LAVmax, LAVmin, LAVpreA, and LVEDV: LACI = LAVmin/LVEDV. Left atrial active ejection fraction (LAAEF) = (LAVpreA − LAVmin)/LAVpreA × 100%. Left atrial passive ejection fraction (LAPEF) = (LAVmax − LAVpreA)/LAVmax × 100%. Here, LAAEF represents left atrial pump function, and LAPEF represents left atrial conduit function.

#### 2.3.3 2D-STI measurement methods

In accordance with the recommendations of the ASE and EACVI, 2D-STI was performed (9). Imaging planes included the apical two-chamber, apical three-chamber, and apical four-chamber views. The average value of all segments was calculated to determine the longitudinal strain at the time of aortic valve closure (AVC), which was used to evaluate LV-GLS (Figures 1D–F). Each wall of the left ventricle was automatically divided into three equal segments (basal, mid, and apical), producing strain values for 18 segments (Figures 1D–F). Subjects with poor image quality, inadequate border tracking, foreshortened views, or missing images were excluded from the analysis.

Left atrial strain was measured using the apical two-chamber and four-chamber views. The zero baseline of the left atrial strain curve was set at left ventricular end-diastole using R–R ECG gating. The endocardial border of the left atrium was manually traced at both left ventricular end-diastole and end-systole, and the average of three consecutive measurements was recorded (Figures 1G–I). Individuals with poor image quality, inadequate border tracking, foreshortened views, or missing images were excluded from the analysis.

The three components of left atrial function were evaluated:

1. Left atrial strain during the reservoir phase (LASR) corresponds to left ventricular systole.
2. Left atrial strain during the conduit phase (LASCD) is measured from mitral valve opening to the onset of the *P* wave.
3. Left atrial strain during the contraction phase (LASCT) is measured from the onset of the *P* wave to mitral valve closure.

These components were compared in the study.

#### 2.3.4 Statistical methods

Statistical analysis was performed using SAS JMP 10.0 software. The normality of data distribution was tested using the Kolmogorov–Smirnov test. Normally distributed data were expressed as mean ± standard deviation (SD). For comparisons among the three groups, one-way analysis of variance (ANOVA) was used. When significant differences were found, pairwise comparisons were conducted using the least significant difference (LSD) method. Non-normally distributed data were described using median and interquartile range (IQR), and group comparisons were made using the Kruskal–Wallis test. Pairwise comparisons of non-normally distributed data were conducted using the Steel–Dwass test. Categorical variables were presented as frequencies and percentages, and comparisons among groups were conducted using the chi-square test. Logistic regression analysis was used to identify factors influencing hypertensive remodeling. For normally distributed variables, Pearson correlation coefficients and corresponding *P*-values were calculated; for non-normally distributed variables, Spearman correlation coefficients and *p*-values were used. Correlation analysis was conducted for variables in the IBSH group. A two-sided *P* < 0.05 was considered statistically significant for all analyses.

## 3 Results

### 3.1 Comparison of clinical data

No significant differences in gender or age were observed among the three groups (all *P* > 0.05). Compared with the control group, both the IBSH and LVH groups showed significantly higher SBP, DBP, blood glucose, blood lipid levels, body mass index (BMI), and B-type natriuretic peptide (BNP) concentrations (all *P* < 0.05). No significant differences were found between the IBSH and LVH groups in these variables (all *P* > 0.05). In addition, the proportion of patients adhering to regular antihypertensive medication use was significantly higher in the IBSH group than in the LVH group (*P* < 0.05) (see Table 1 for details).

**Table 1:** General clinical data of control group, LVH group, and IBSH group [($\bar{x}\pm s$), *n* (%), M (Q25, Q75)].

Index	Controls (*n* = 70)	LVH (*n* = 70)	IBSH (*n* = 70)	*F*/*H*	*P*
Gender (male) [example (%)]	37 (52.86%)	40 (57.14%)	39 (55.71%)	0	1.000
Age	56.49 ± 11.87	58.81 ± 8.53	59.67 ± 10.85	1.722	0.181
Hypertensive years	–	8.00 (3.75, 14.00)	9.00 (3.00, 15.25)	0.071	0.943
Take medication regularly (yes)	–	40 (57.14%)	57 (81.43%)	9.700	0.002
BMI	23.00 (21.00, 26.00)	27.00 (24.00, 29.00)^a^	26.00 (23.75, 28.00)^a^	22.757	<0.001
FBG	5.40 (5.10, 5.72)	5.75 (5.30, 6.73)^a^	5.80 (5.30, 6.23)^a^	21.22	<0.001
TG	1.12 (0.68, 2.32)	1.51 (1.04, 2.15)	1.46 (1.02, 2.04)	0.294	0.071
TCHO	4.00 (3.32, 4.54)	4.45 (3.62, 5.11)^a^	4.06 (3.43, 4.60)	9.587	0.008
HDL	1.24 (0.96, 1.44)	0.97 (0.83, 1.12)^a^	1.00 (0.87, 1.14)^a^	31.875	<0.001
LDL	2.46 (2.08, 2.95)	2.85 (2.36, 3.25)^a^	2.62 (2.10, 3.12)	8.825	0.012
SBP	120.00 (115.00, 125.00)	143.50 (127.00, 157.75)^a^	139.00 (129.75, 150.25)^a^	81.841	<0.001
DBP	76.00 (71.75, 80.00)	86.00 (76.00, 102.50)^a^	84.50 (76.00, 90.00)^a^	35.280	<0.001
BNP	41.70 (23.33, 75.58)	78.95 (33.73, 144.75)^a^	75.80 (33.83, 138.25)^a^	18.705	<0.001

BMI, ballistic missile intercept; FBG, fasting blood glucose; TG, triglyceride; TCHO, total cholesterol; HDL, high-density lipoprotein; LDL, low-density lipoprotein; SBP, systolic blood pressure; DBP, diastolic blood pressure; BNP, brain natriuretic peptide.

a Versus controls, *P* < 0.05.

b Versus LVH, *P* < 0.05.

Multivariate regression analysis of SBP, DBP, blood glucose, blood lipids, BMI, and BNP identified fasting blood glucose (FBG) as a common risk factor for both the IBSH and LVH groups [odds ratio (OR) = 3.204, 95% confidence interval (CI): 1.454–7.061; OR = 5.080, 95% CI: 2.012–12.824]. BMI was identified as an independent risk factor for the LVH group (OR = 1.226, 95% CI: 1.087–1.382), while high-density lipoprotein (HDL) was a common protective factor for both the IBSH and LVH groups (OR = 0.091, 95% CI: 0.019–0.448; OR = 0.031, 95% CI: 0.005–0.185) (all *P* < 0.05\) (see Table 2 for details).

**Table 2:** Logistic regression analysis of factors influencing left ventricular remodeling.

Index	*β*	SE	Wald *χ*^2^	*P*	OR (95%CI)
IBSH					
Constant	−6.916	2.802	6.091	0.014	
BMI	0.122	0.063	3.785	0.052	1.130 (0.999–1.279)
FBG	1.164	0.403	8.341	0.004	3.204 (1.454–7.061)
HDL	−2.392	0.811	8.712	0.002	0.091 (0.019–0.448)
LVH					
Constant	−10.653	3.292	10.473	0.002	
BMI	0.203	0.061	11.047	<0.001	1.226 (1.087–1.382)
FBG	1.625	0.472	11.832	<0.001	5.080 (2.012–12.824)
HDL	−3.460	0.906	14.601	<0.001	0.031 (0.005–0.185)

After conducting a comparative analysis of the antihypertensive medications used by patients in the IBSH group and the LVH group, the statistical test results indicated that there was no statistically significant difference in the types or regimens of antihypertensive drugs between the two groups of patients (*P* > 0.05) (see Table 3 for details).

**Table 3:** Analysis of differences in antihypertensive drugs between the IBSH group and the LVH group [($\bar{x}\pm s$), M (Q25, Q75)].

Hypertension medication	LVH (*n* = 70)	IBSH (*n* = 70)	*χ* ^2^	*P*
β-Blockers + CCB + diuretics	3 (4.3)	1 (1.4)	0.257	0.612
ARB + CCB + diuretics	13 (18.6)	12 (17.1)	0.049	0.825
β-Blockers + ARBs	18 (25.7)	15 (21.4)	0.357	0.550
β-Blockers + CCB	4 (5.7)	2 (2.9)	0.174	0.676
ARB + diuretics	2 (2.9)	3 (4.3)	0.000	>0.999
CCB + diuretics	4 (5.7)	5 (7.1)	0.000	>0.999
β-Blockers	3 (4.3)	2 (2.9)	0.000	>0.999
Diuretics	2 (2.9)	4 (5.7)	0.174	0.676
CCB	9 (12.9)	12 (17.1)	0.504	0.478
ARB	12 (17.1)	14 (20.0)	0.189	0.664

β-Blockers, beta-adrenoceptor antagonist; ARBs, angiotensin II receptor blockers; CCB, calcium channel blockers.

a Versus LVH, *P* < 0.05.

### 3.2 Comparison of left ventricular cavity size and function

**Figure 2:**
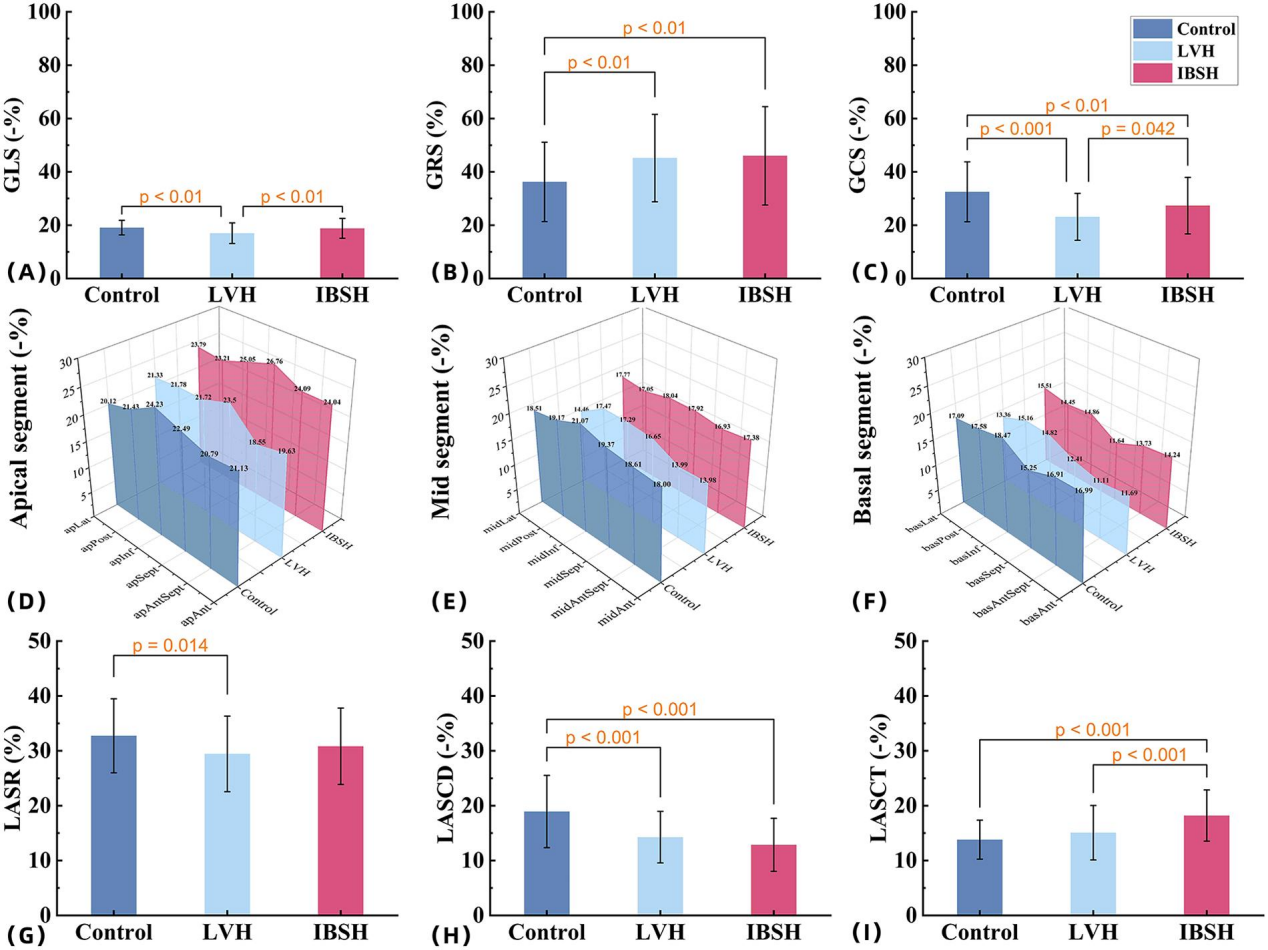
**(A–C)** The comparisons among the control group, LVH group, and IBSH group for GLS, GRS, and GCS, respectively. **(D–F)** The comparisons among the control group, LVH group, and IBSH group for the apical segment, mid-segment, and basal segment, respectively. **(G–I)** The comparisons among the control group, LVH group, and IBSH group for LASR, LASCD, and LASCT, respectively. All abbreviations are consistent with those in Tables 4 and 6.

Compared with the control group, both the IBSH and LVH groups showed increased IVSD, LVSBD, RWT, and LVMI (*P* < 0.01). In comparisons between the IBSH and LVH groups, the IBSH group had significantly lower LVSd, LVSBD, RWT, and LVMI (*P* < 0.01). Both the IBSH and LVH groups exhibited reduced GLS and GCS compared with the control group, with a statistically significant reduction in GLS in the LVH group (*P* < 0.01). GRS was increased in both groups (*P* < 0.01). Compared with the LVH group, the IBSH group had higher GLS, GCS, and GRS, with a significant difference observed in GLS (*P* < 0.01). Compared with the control group, the IBSH group showed significantly higher E and E/A ratio, while atrial contraction velocity (A) was significantly lower (*P* < 0.01). In intergroup comparisons, the IBSH group had significantly higher A values and lower E/A ratios compared with the LVH group (*P* < 0.01) (see Table 4 and Figures 2F for details).

**Table 4:** Analysis results of left ventricular size and function difference among the control group, LVH group, and IBSH group [($\bar{x}\pm s$), M (Q25, Q75)].

Index	Controls (*n* = 70)	LVH (*n* = 70)	IBSH (*n* = 70)	*F*/*H*	*P*
LVSd	0.80 (0.80, 0.83)	1.20 (1.20, 1.30)^a^	1.00 (0.90, 1.00)^a^^,^^b^	174.604	<0.001
LVSBD	0.80 (0.80, 0.83)	1.20 (1.20, 1.30)^a^	1.50 (1.40, 1.60)^a^^,^^b^	190.153	<0.001
LVPWd	0.80 (0.70, 0.80)	1.20 (1.18, 1.20)^a^	0.90 (0.80, 1.00)^a^^,^^b^	166.456	<0.001
RWT	0.34 (0.33, 0.36)	0.51 (0.49, 0.52)^a^	0.41 (0.38, 0.43)^a^^,^^b^	176.192	<0.001
LVEDV	103.00 (95.00, 108.00)	103.00 (97.25, 113.00)	95.00 (90.00, 100.50)^a^^,^^b^	22.476	<0.001
LVESV	39.00 (37.75, 41.00)	41.50 (40.00, 43.00)^a^	38.00 (37.00, 40.00)^a^^,^^b^	49.863	<0.001
LVMI	79.98 (75.25, 85.22)	139.38 (133.86, 145.18)^a^	101.41 (91.73, 111.36)^a^^,^^b^	175.041	<0.001
LVEF	62.00 (59.00, 63.00)	60.00 (59.00, 62.00)^a^	60.00 (59.00, 62.00)^a^	11.300	0.004
GLS	−19.11 ± 2.72	−16.90 ± 4.01^a^	−18.89 ± 3.69^b^	8.358	<0.001
GCS	−32.61 (−40.24, −21.40)	−22.59 (−29.24, −15.75)^a^	−25.59 (−33.65, −17.73)	22.908	<0.001
GRS	34.24 (23.13, 46.64)	40.56 (31.06, 52.11)	45.92 (32.34, 57.34)^a^	11.006	0.004
E	0.79 (0.67, 0.90)	0.66 (0.61, 0.76)^a^	0.62 (0.55, 0.72)^a^	36.821	<0.001
A	0.67 (0.55, 0.78)	0.74 (0.67, 0.82)^a^	0.81 (0.72, 0.88)^a^^,^^b^	29.336	<0.001
E/A	1.36 (0.84, 1.55)	0.86 (0.81, 0.92)^a^	0.76 (0.68, 0.83)^a^^,^^b^	60.548	<0.001
E/e′	8.66 (7.49, 9.90)	9.18 (8.73, 10.13)	9.29 (8.19, 10.25)	5.782	0.056
Peak TR velocity	2.10 (1.91, 2.32)	2.32 (2.10, 2.45)^a^	2.24 (2.00, 2.4)	13.725	0.001

LVSd, left ventricular septal end-diastolic thickness; LVSBD, left ventricular septal basal-segment end-diastolic thickness; LVPWd, left ventricular posterior wall end-diastolic thickness; LVEDV, left ventricular end-diastolic volume; LVESV, left ventricular end-systolic volume; RWT, relative wall thickness; LVEF, left ventricular ejection fraction; LVMI, left ventricular mass index; GLS, global longitudinal strain; GCS, global circumferential strain; GRS, global radial strain; E, early diastolic transmitral inflow velocity; A, after diastolic transmitral inflow velocity.

a Versus controls, *P* < 0.05.

b Versus LVH, *P* < 0.05.

In the 18-segment strain analysis of the left ventricle, compared with the control group, myocardial strain in both the LVH and IBSH groups showed a progressive decrease from the apex to the base. A significant reduction was observed in the basal segments, especially in the anterior and posterior septum of the IBSH group (see Table 5 and Figures 2D-F for details).

**Table 5:** Analysis results of 18-segment differences of left ventricular strain in control group, LVH group, and IBSH group [($\bar{x}\pm s$), M (Q25, Q75)].

Index	Controls (*n* = 70)	LVH (*n* = 70)	IBSH (*n* = 70)	*F*/*Z*	*P*
apAnt	−21.13 ± 6.23	−19.63 ± 8.56^a^	−24.04 ± 8.56	5.638	0.004
apAntSept	−20.79 (−24.61, −16.28)	−18.55 (−26.56, −13.22)	−24.09 (−29.26, −15.53)	5.730	0.057
apSept	−22.49 ± 5.51	−23.50 ± 6.20^a^	−26.76 ± 6.27^b^	9.600	<0.001
apInf	−24.23 ± 5.10	−21.72 ± 7.61^a^	−25.05 ± 7.42	4.544	0.012
apPost	−21.43 ± 4.49	−21.78 ± 8.68	−23.21 ± 8.35	1.129	0.325
apLat	−20.12 ± 7.05	−21.33 ± 6.79^b^	−23.79 ± 7.22^b^	4.916	0.008
midAnt	−18.00 ± 5.60	−13.98 ± 7.13^a^^,^^b^	−17.38 ± 7.27	7.262	<0.001
midAntSept	−18.61 ± 5.71	−13.99 ± 6.94^a.b^	−16.93 ± 7.64	8.254	<0.001
midSept	−19.37 ± 3.04	−16.65 ± 4.53^b^	−17.92 ± 4.44	7.873	<0.001
midInf	−21.07 ± 4.39	−17.29 ± 5.15^b^	−18.04 ± 4.46^b^	12.78	<0.001
midPost	−19.17 ± 4.57	−17.47 ± 5.35	−17.05 ± 5.13^b^	3.469	0.033
midLat	−18.51 (−21.72, −13.85)	−14.46 (−18.62, −11.32)^b^	−17.77 (−21.43, −11.12)	8.783	0.012
basAnt	−16.99 (−19.83, −14.45)	−11.69 (−15.11, −6.38)^b^	−14.24 (−17.90, −9.56)^b^	26.846	<0.001
basAntSept	−16.91 ± 5.49	−11.11 ± 6.18^b^	−13.73 ± 6.93^b^	15.204	<0.001
basSept	−15.25 (−17.87, −12.98)	−12.41 (−15.07, −9.64)^b^	−11.64 (−15.07, −8.78)^b^	37.110	<0.001
basInf	−18.47 ± 4.55	−14.82 ± 4.56^b^	−14.86 ± 4.45^b^	15.046	<0.001
basPost	−17.58 ± 5.24	−15.16 ± 4.82^b^	−14.45 ± 5.00^b^	7.471	<0.001
basLat	−17.09 (−20.48, −13.07)	−13.36 (−16.92, −8.33)^b^	−15.51 (−19.50, −10.32)	14.526	<0.001

a Versus LVH, *P* < 0.05.

b Versus controls, *P* < 0.05.

### 3.3 Comparison of left atrial size and function

Compared with the control group, both the IBSH and LVH groups showed significant increases in LAVmax, LAVmin, LAVpreA, LAVI, and LACI (all *P* < 0.01), while LATEF was significantly reduced (*P* < 0.01). No significant differences were observed between the IBSH and LVH groups (*P* > 0.05). Compared with the control group, left atrial strain during the reservoir phase (LASR) and LAS during the conduit phase (LASCD) were reduced in both IBSH and LVH groups, while left atrial strain during the contraction phase left atrial contraction strain (LASCT) was increased in the IBSH group. LASCD and LASCT showed statistically significant differences (all *P* < 0.01). In comparisons between IBSH and LVH groups, LASCT was significantly higher in the IBSH group (*P* < 0.01) (see Table 6 and Figure 2I for details).

**Table 6:** Analysis results of the difference of left atrial size and function among different groups in the control group, LVH group, and IBSH group [($\bar{x}\pm s$), M (Q25, Q75)].

Index	Controls (*n* = 70)	LVH (*n* = 70)	IBSH (*n* = 70)	*F*/*H*	*P*
LAVmax	32.00 (27.00, 40.00)	46.00 (38.75, 59.00)^a^	41.50 (35.00, 48.25)^a^	42.248	<0.001
LAVmin	11.00 (9.00, 14.00)	17.00 (13.75, 22.00)^a^	15.50 (11.00, 18.00)^a^	45.738	<0.001
LAVpreA	22.00 (18.00, 30.25)	35.00 (26.75, 38.75)^a^	30.00 (24.00, 36.50)^a^	35.729	<0.001
LATEF	66.00 (64.00, 68.00)	63.50 (60.75, 67.00)^a^	64.00 (60.00, 66.25)^a^	18.409	<0.001
LAAEF	51.00 (43.75, 58.25)	47.00 (41.00, 52.25)	49.00 (42.00, 55.00)	4.442	0.109
LAPEF	29.69 ± 14.25	29.53 ± 9.65	28.96 ± 10.23	0.077	0.926
LAVI	20.73 (18.64, 25.00)	24.93 (21.41, 28.36)^a^	23.09 (19.54, 29.41)	17.701	<0.001
LACI	11.00 (9.00, 13.00)	17.00 (13.00, 22.00)^a^	15.00 (12.00, 20.00)^a^	45.76	<0.001
LASR	32.74 ± 6.74	29.44 ± 6.89^a^	30.84 ± 6.95	4.077	0.018
LASCD	18.00 (14.00, 18.00)	14.00 (11.00, 16.25)^a^	12.50 (10.00, 15.25)^a^	37.156	<0.001
LASCT	13.00 (11.00, 16.00)	13.00 (11.00, 18.25)	18.00 (14.00, 21.00)^a^^,^^b^	37.732	<0.001

LAVmax, left atrial volume max; LAVmin, left atrial volume min; LAVpreA, left atrial volume preA; LATEF, left atrial total ejection fraction; LAAEF, left atrial active ejection fraction; LAVI, left atrial volume index; LACI, left atrioventricular coupling index; LASR, left atrial reservoir strain; LASCD, left atrial conduit strain; LASCT, left atrial contraction strain.

a Versus controls, *P* < 0.05.

b Versus LVH, *P* < 0.05.

### 3.4 Correlation analysis among variables in the IBSH group

**Figure 3:**
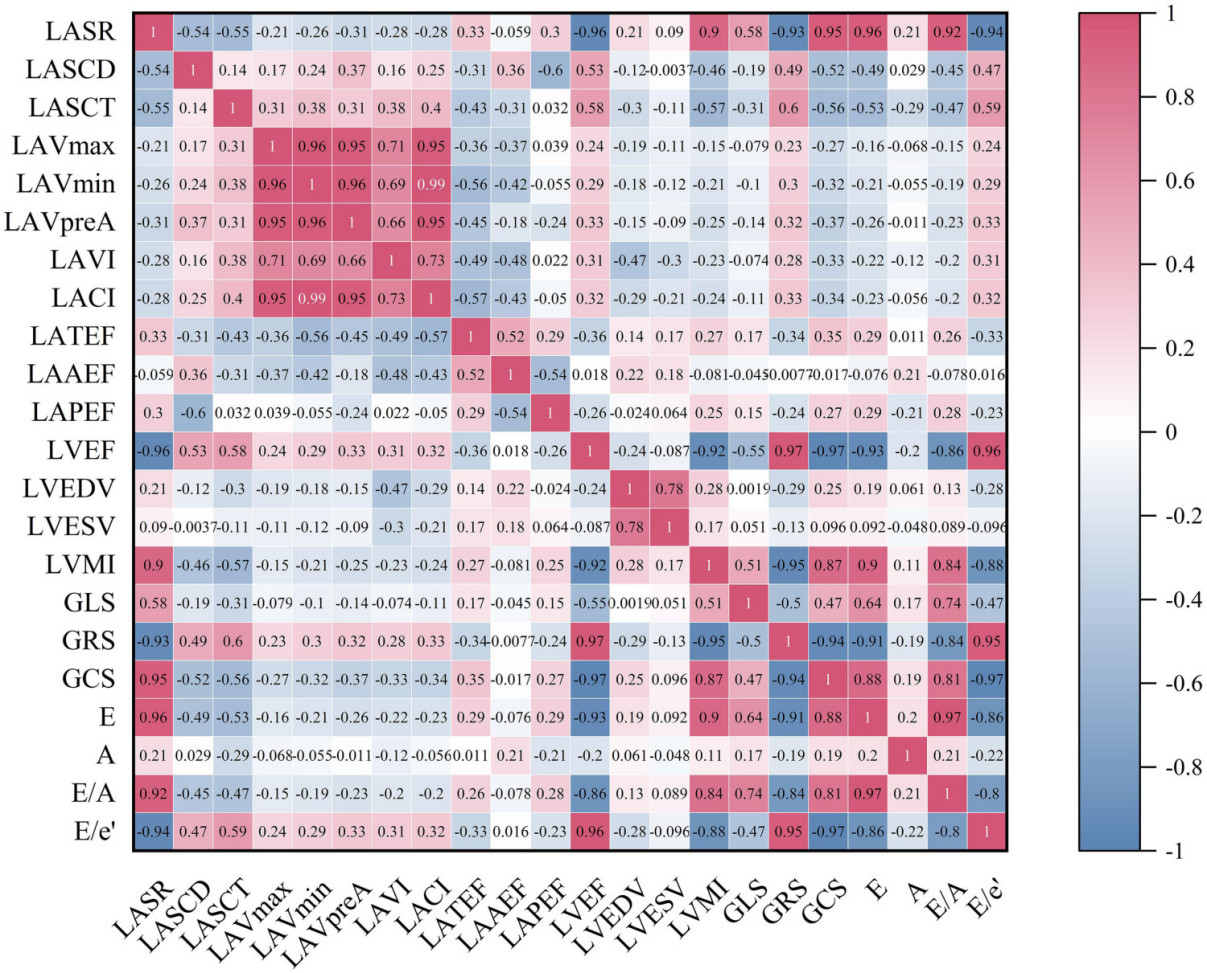
The heatmap represents the correlation analysis among various variables in the IBSH group. The color shading in each cell corresponds to the magnitude and direction of the correlation coefficient, with red indicating a positive correlation and blue indicating a negative correlation. The numerical value within each cell represents the bivariate correlation coefficient. All abbreviations are consistent with those in Tables 4 and 6.

In the IBSH group, LVMI was negatively correlated with LVEF, GRS, and GCS (*r* = −0.92, *r* = −0.95, *r* = −0.87; all *P* < 0.05). LACI was positively correlated with LAVI (*r* = 0.73, *P* < 0.05). LASR was positively correlated with GLS, E, and E/A but negatively correlated with the ratio of early diastolic mitral inflow velocity to early diastolic mitral annular velocity (E/e′) (*r* = 0.58, *r* = 0.96, *r* = 0.92, *r* = −0.94; all *P* < 0.05). LASCD was negatively correlated with E and E/A and positively correlated with E/e′ (*r* = −0.49, *r* = −0.45, *r* = 0.47; all *P* < 0.05). LASCT was also negatively correlated with E and E/A and positively correlated with E/e′ (*r* = −0.53, *r* = −0.47, *r* = 0.59; all *P* < 0.05) (see Figure 3 for details).

## 4 Discussion

Our study confirmed that both the LVH and IBSH groups exhibited significant abnormalities in blood pressure, blood glucose, blood lipids, BMI, and BNP levels. Multivariate regression analysis indicated that elevated blood glucose was a common risk factor for both groups. BMI emerged as an independent risk factor specifically in the LVH group, whereas HDL was identified as a protective factor common to both the IBSH and LVH groups. These factors, along with hypertension, had varying effects on left ventricular remodeling, consistent with previous studies (11, 12).

Additionally, LVMI, a marker of left ventricular remodeling, was significantly increased in both the IBSH and LVH groups, with a greater increase observed in the LVH group. Stratified analysis showed that the proportion of patients regularly using antihypertensive medication was higher in the IBSH group than that in the LVH group, suggesting that consistent use of antihypertensive drugs may influence ventricular remodeling in the IBSH group. However, no statistically significant difference was observed between the two groups in terms of the types or regimens of antihypertensive drugs used. This observation has not been previously reported and requires further research for confirmation.

Indicators of early left ventricular systolic function, including GLS and GCS, showed a decreasing trend in both the IBSH and LVH groups. Long-term elevation in afterload resulted in varying degrees of systolic dysfunction in both groups, with more severe impairment in the LVH group. The IBSH group showed a significant increase in GRS due to compensatory mechanisms (see Figure 2B), which may have contributed to the preservation of systolic function, consistent with previous findings (13).

In the 18-segment analysis of systolic mechanics, the IBSH group showed increased myocardial strain only in the apical segment, while the other segments exhibited reduced strain, with the most notable decrease in the anterior and posterior septum of the basal segment (see Figures 2D–F). In contrast, the LVH group demonstrated extensive impairment across all segments. This pattern is related to the uneven distribution of wall stress in hypertension, where stress progressively increases from the apex to the base (14). These findings suggest that damage in the basal segments may be partially compensated for by functional reserve in the apical segment. This redistribution is closely associated with myocardial remodeling and appears more evident in IBSH patients (15).

Previous studies have shown that left atrial size is closely related to left ventricular filling pressure (16). LACI is a crucial metric for evaluating the functional coupling between the left atrium and the left ventricle. Studies have reported that a higher LACI reflects greater imbalance between left atrial and left ventricular volumes, indicating more severe impairment of coupling function (17). In this study, LACI was significantly increased in both the IBSH and LVH groups compared with the control group. However, myocardial impairment was less severe in the IBSH group than in the LVH group, suggesting that although atrioventricular coupling was impaired in IBSH, compensatory mechanisms may have helped preserve myocardial function.

The findings of this study indicate that both the IBSH and LVH groups exhibited significantly lower LASR and LASCD in the LA compared with the control group (18). However, LASCT showed a compensatory increase in the IBSH group, while it was significantly lower in the LVH group compared with the IBSH group. Otani et al. (19) reported that LASCT increases compensatorily in patients with mild to moderate diastolic dysfunction (see Figures 2G–I), which is consistent with the compensatory increase in left ventricular GRS. Therefore, the enhancement of LASCT may function as a regulatory mechanism for impaired left ventricular filling in the IBSH group (20).

This study found that the reduction in LASR was associated with impaired GLS (see Figure 3), indicating that the decline in left atrial function is linked to the secondary effects of persistently elevated left ventricular volume and pressure overload (21). As a result, left ventricular diastolic function was impaired in both the LVH and IBSH groups. Moreover, LASR, LASCD, and LASCT in the IBSH group were correlated with E, E/A, and E/e′, which are indicators of left ventricular diastolic function (see Figure 3), further suggesting that diastolic dysfunction in the IBSH group may contribute to reduced left atrial function.

## 5 Limitations

This study has certain limitations. Firstly, it is a single-center design, making it difficult to avoid inherent selection bias. Secondly, it may be subject to admission rate bias and misclassification bias. Finally, the sample size is relatively limited, and future validation through longer-term follow-up and larger-scale samples is still required.

## 6 Conclusions

This study reveals a potential association between regular antihypertensive medication use and cardiac remodeling in patients with IBSH, offering new research directions for understanding the pathophysiological mechanisms of hypertension combined with myocardial hypertrophy. By analyzing changes in global and regional left atrioventricular myocardial function, the study identified the basal septal segment as the most significantly impaired region and confirmed the existence of a compensatory redistribution mechanism in myocardial mechanics. This finding enriches research in the field of cardiac remodeling and provides an important theoretical basis for further exploration of hypertensive heart disease and myocardial compensatory mechanisms.

## Data Availability

The original contributions presented in the study are included in the article/Supplementary Material, further inquiries can be directed to the corresponding author/s.

## Glossary

2D-STI: two-dimensional speckle-tracking imaging
ASE: American Society of Echocardiography
A: late diastolic transmitral inflow velocity
BMI: ballistic missile intercept
BNP: brain natriuretic peptide
DBP: diastolic blood pressure
E: early diastolic transmitral inflow velocity
E′: early diastolic transmitral inflow
EAE: European Association of Echocardiography
EACVI: European Association of Cardiovascular Imaging
FBG: fasting blood glucose
GLS: global longitudinal strain
GCS: global circumferential strain
GRS: global radial strain
HDL: high-density lipoprotein
IBSH: isolated basal septal hypertrophy
LACI: left atrioventricular coupling index
LDL: low-density lipoprotein
LV: left ventricle
LA: left atrial
LVH: left ventricular hypertrophy
LVMI: left ventricular mass index
LAVI: left atrial volume index
LACI: left atrioventricular coupling index
LASR: left atrial reservoir strain
LASCD: left atrial conduit strain
LASCT: left atrial contraction strain
LVEF: left ventricular ejection fraction
LVSBD: left ventricular septal basal-segment end-diastolic thickness
LVSd: left ventricular septal end-diastolic thickness
LVDd: left ventricular diameter diastolic
LVDs: left ventricular diameter systolic
LVPWd: left ventricular posterior wall end-diastolic thickness
LVEDV: left ventricular end-diastolic volume
LVESV: left ventricular end-systolic volume
LVEDd: left ventricle end-diastolic diameter
LAAD: left atrial end-diastolic dimension
LAVmax: left atrial volume max
LAVmin: left atrial volume min
LAVpreA: left atrial volume preA
LATEF: left atrial total ejection fraction
LAAEF: left atrial active ejection fraction
LAPEF: left atrial passive ejection fraction
RWT: relative wall thickness
SBP: systolic blood pressure
TG: triglyceride
TCHO: total cholesterol
TDI: tissue Doppler imaging.
